# Cuttlefish-Bone-Derived Hybrid Composite Scaffolds for Bone Tissue Engineering

**DOI:** 10.3390/nano15030196

**Published:** 2025-01-26

**Authors:** Vignesh Raj Sivaperumal, Sutha Sadhasivam, Ramalingam Manikandan, Ilanchezhiyan Pugazhendi, Saravanan Sekar, Youngmin Lee, Sejoon Lee, Sankar Sekar

**Affiliations:** 1 Department of Pharmaceutical Technology, Dhanalakshmi Srinivasan Engineering College (Autonomous), Perambalur 621 212, Tamil Nadu, India; vigneshraj.s@dsengg.ac.in; 2 Department of Chemistry, CMS College of Engineering, Ernapuram, Namakkal 637 003, Tamil Nadu, India; suthaasridhar@gmail.com; 3 Department of Analytical Chemistry, University of Madras, Chennai 600 025, Tamil Nadu, India; maniiichem@pusan.ac.kr; 4 Quantum-Functional Semiconductor Research Center, Dongguk University-Seoul, Seoul 04620, Republic of Korea; ilancheziyan@dongguk.edu (I.P.); ymlee@dongguk.edu (Y.L.); sejoon@dongguk.edu (S.L.); 5 Department of Mechanical Engineering, K. Ramakrishnan College of Technology, Trichy 621 112, Tamil Nadu, India; nanosaran007@gmail.com; 6 Division of System Semiconductor, Dongguk University-Seoul, Seoul 04620, Republic of Korea

**Keywords:** cuttlefish bone, hydrothermal, scaffolds, biocompatibility, hydroxyapatite

## Abstract

Current investigations into the fabrication of innovative biomaterials that stimulate cartilage development result from increasing interest due to emerging bone defects. In particular, the investigation of biomaterials for musculoskeletal therapies extensively depends on the development of various hydroxyapatite (HA)/sodium alginate (SA) composites. Cuttlefish bone (CFB)-derived composite scaffolds for hard tissue regeneration have been effectively illustrated in this investigation using a hydrothermal technique. In this, the HA was prepared from the CFB source without altering its biological properties. The as-developed HA nanocomposites were investigated through XRD, FTIR, SEM, and EDX analyses to confirm their structural, functional, and morphological orientation. The higher the interfacial density of the HA/SA nanocomposites, the more the hardness of the scaffold increased with the higher applied load. Furthermore, the HA/SA nanocomposite revealed a remarkable antibacterial activity against the bacterial strains such as *E. coli* and *S. aureus* through the inhibition zones measured as 18 mm and 20 mm, respectively. The results demonstrated a minor decrease in cell viability compared with the untreated culture, with an observed percentage of cell viability at 97.2% for the HA/SA nanocomposites. Hence, the proposed HA/SA scaffold would be an excellent alternative for tissue engineering applications.

## 1. Introduction

The rising health care expenses and continuous development of medical systems around the globe is a major concern due to tissue and organ damage, which significantly affects the lifespan of individuals [1,2,3,4]. Generally, tissue engineering encompasses the development of repair, sustainability, increased activity, or improvement in the life quality of an individual by the restoration or replacement of tissue and organs employing biocompatible compounds [5,6]. Due to its unique properties such as physical and chemical stability and biological activity, porous structures enhance the tissue regeneration and healing ability of the artificially developed biomaterials [7]. Hydroxyapatite (HA) has been widely studied as a biologically active material that stimulates allografts and xenograft bone restoration due to its physical, chemical, biological, and mechanical properties, similar to that of human bone [8,9,10]. On the other hand, the replacement of tissues during implantation in the human body leads to permanent failure, which may be due to foreign body reaction, microbial infections, aseptic loosening, and wear resistance [11,12,13].

During the last few decades, the fabrication of HA has been performed through various chemical techniques, and this commercial synthesis method has several disadvantages like high fabrication cost, environmental pollution, etc. [14,15,16]. To minimize the use of chemical precursors, the development of calcium from biogenic waste sources has shown huge interest in the biomedical field during the last few years [17]. Generally, the calcium source has been extracted from various biogenic sources such as bovine bone, porcine, fish scales, fish bones, and other shells [9,18,19,20]. For various biomedical applications, the fabrication of HA from biogenic waste materials exhibits several benefits including enhanced mechanical properties, improved biocompatibility, minimal toxic nature to the cells, and remarkable biodegradability [18]. Furthermore, HA has been extracted from biogenic waste materials using different fabrication techniques such as hydrothermal, ultrasonication, co-precipitation, thermal decomposition, wet chemical, etc. [21]. Recently, the extraction of calcium sources from cuttlefish bone (CFB) has shown a unique architecture, high stiffness, and high permeability [22,23,24]. In addition, the major presence of aragonite phase in the lamellar region can be hydrothermally transformed into HA without affecting the micro-structural arrangements, porosity, and interconnecting microstructures [25]. Even though HA exhibits enhanced biocompatibility, its low fracture toughness limits its applications in the biomedical field [26]. To overcome this, various polymeric substitutions have been performed in HA to achieve a flexible structure formation with improved mechanical, antibacterial, and biocompatible materials [27].

Further, the formation of inorganic composite materials along with biopolymers exhibit enhanced biological performances [28]. A wide variety of natural and synthetic polymers such as poly lactic acid, poly acrylic acid, chitosan, sodium alginate (SA), etc., can be utilized as a composite material for tissue engineering and drug delivery applications to avoid infections and pain [29,30,31,32]. In this, the SA biopolymer reveals extensive biocompatibility, biodegradability, better mechanical properties, and a non-toxic nature with enhanced microbial resistance, leading it to be a potential biopolymer for the fabrication of scaffolds for biomedical applications [33]. The incorporation of the polymeric material in HA was found to have potential for the improvement of physico-chemical and biological characteristics such as biodegradability, water absorption ability, and osteoinductive and osteoconductive properties [34]. Generally, the human body has the ability to reconstruct small damage but requires additional treatment for larger bone defects. Even though it possesses healing ability, some diseases such as cancer, osteoporosis, fracture, and infections require additional treatment for the recovery of bone tissue. All of the above background knowledge promoted us to fabricate and investigate cuttlefish-bone-derived HA combined with SA hybrid nanocomposite for improved antibacterial, cell viability, and nanomechanical strength evaluation for application in bone tissue engineering.

In the present work, the preparation of HA was obtained through the hydrothermal method from CFB at an optimized sintering temperature at 1200 °C. The prepared HA nanoparticles formed a scaffold with SA for tissue engineering applications. The prepared nanoparticles and the HA/SA scaffold were characterized through the XRD, FTIR, SEM, and EDX analyses. Furthermore, the bacterial resistance of the as-prepared nanocomposite was analyzed against the Gram-positive and Gram-negative bacterial strains such as *S. aureus* and *E. coli,* employing the disc diffusion method. The cytotoxicity investigation of the as-prepared nanocomposite was investigated through MTT assay using the MG 63 human osteosarcoma cell culture.

## 2. Materials and Methods

### 2.1. Preparation of HA Nanoparticles

**Figure 1:**
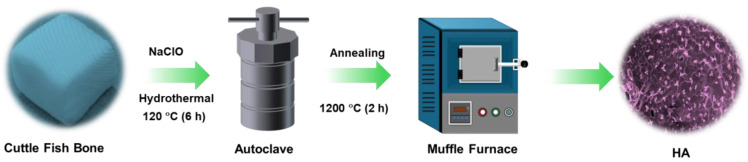
A schematic illustration of the fabrication and application of HA/SA from cuttlefish bone derived from high-temperature hydrothermal treatment.

The cuttle fish bone (CFB) was collected from the local market and washed with distilled water several times for the removal of unwanted debris and protein substances, and then the bone was cut into small pieces from the lamellar region in the size range of 1 × 1.5 × 1 cm^3^, as shown in Figure 1. After that, the bone pieces were soaked in sodium hypochlorite (13% NaClO) for 12 h to remove the organic residues and washed 3 times with distilled water. Then, the washed CFB pieces were crushed into small pieces using a mechanical grinder and introduced into a 0.6 M diammonium hydrogen phosphate solution and stirred for 3 h in a magnetic agitator. After that, the obtained mixture was hydrothermally treated at 180 °C for 48 h. After completion of the reaction, the obtained sample was filtered, and the filtrate was oven dried at 70 °C for 6 h to obtain the dry powder. Finally, the obtained powder was calcined at 1200 °C for 2 h in a tubular furnace to obtain the final HA nanoparticles. The as-developed HA nanoparticles were stored and further used for the scaffold preparation.

### 2.2. Preparation of the HA/SA Scaffold

In this, the HA/SA scaffold was fabricated using a solvent casting technique. For the preparation of the scaffold, 2 wt.% of the SA was dissolved in 100 mL distilled water, and the mixture was allowed to be stirred for 2 h at 38 °C. After complete dissolution of the SA, the 1 wt.% of HA nanoparticles was blended, and the mixture was continually stirred for 24 h. Finally, the HA/SA composite mixture was poured into a sterile Petri dish and then allowed to dry in an air oven at 100 °C for 12 h. The obtained dried scaffolds were collected and used for further characterizations.

### 2.3. Material Characterizations

The functional group of the HA and HA/SA composites was investigated through Fourier transform infrared spectroscopy (FTIR; Spectrum 100; Perkin Elmer, USA) through the KBr pellet technique. An X-ray diffractometer (XRD; X’Pert PRO; PANalytical, The Netherlands) using CuKα as a radiation source in the 2θ range of 10° to 80° was used to analyze the phase purity and crystalline nature of the samples. Scanning electron microscopy and energy dispersive X-ray analysis (SEM-EDX, SIGMA-VP, O-XPORT, Germany) were utilized to investigate the surface morphology and composition of the HA and HA/SA scaffolds. Swelling and degradation of the CFB, HA, and HA/SA were analyzed by immersing the composite in the stimulated body fluid (SBF) solution for 14 days under humidified conditions.

### 2.4. Nanoindentation

A Berkovich diamond indenter fitted to a TriboIndenter (Hysitron, Minneapolis, MN, USA) was used to examine the mechanical properties of the HA/SA scaffold. Using a higher applied peak load of 4–16 µN, the load–displacement pattern was continually monitored. After 10 s of holding the imposed load, it was unloaded at a ratio of 0.2 s µN. The tangent slope derived from the indentation study and the Oliver–Pharr relation were used to determine the hardness (H) and Young’s modulus (E) values [35].

### 2.5. Antibacterial Activity

The antibacterial capabilities of the CFB, HA, and HA/SA scaffolds were evaluated against harmful microorganisms including *E. coli* and *S. aureus* at a dosage of 50 μL using the disc diffusion technique. The freshly cultivated microbial culture was inoculated on the plates and kept at 37 °C. After inoculation, a 4 mm disc was inserted into each plate at the identical position. In addition, the plates were loaded with the HA and HA/SA composites and left overnight at 37 °C. The degree of resistance for every bacterial strain was determined through measuring the clear area that developed around the disk. The average area of zone formation was measured in triplicate for each bacterial strain [36].

### 2.6. Cell Culture Analysis

The human osteosarcoma MG 63 cell culture was used to examine the biocompatibility of the synthesized HA and SA/HA composite. For the biocompatibility analysis, 1.5 × 10^4^ cell mL^−1^ cell cultures were seeded in 24-well plates using MG 63 human osteosarcoma cell cultures. The cultures were cultivated in Eagle’s media with 10% fetal bovine serum in an environment of 5% carbon dioxide. Phenazine methosulphate and the tetrazolium compound 3-(4,5-dimethylthiazol-2-yl)-5-(3-carboxymethoxy-phenyl)-2-(4-sulfophenyl)-2H-tetrazolium were used to analyze the cells. After 72 h, the samples were taken out of the wells, and the culture was cleaned using a phosphate buffer saline solution. Using a spectrophotometer, the UV absorbance at 570 nm was measured, and the average percentage of viable cells was investigated in duplicate.

### 2.7. Statistical Analysis

Using the one-way ANOVA Dunnett’s multi-comparison analysis using GraphPad Prism version 8 software (GraphPad Software, San Diego, CA, USA), a statistical investigation was carried during the evaluation of antibacterial, swelling, degradation, and cell viability for the CFB, HA, and HA/SA nanocomposites, and the significant variations are indicated using *p*-values.

## 3. Results and Discussion

**Figure 2:**
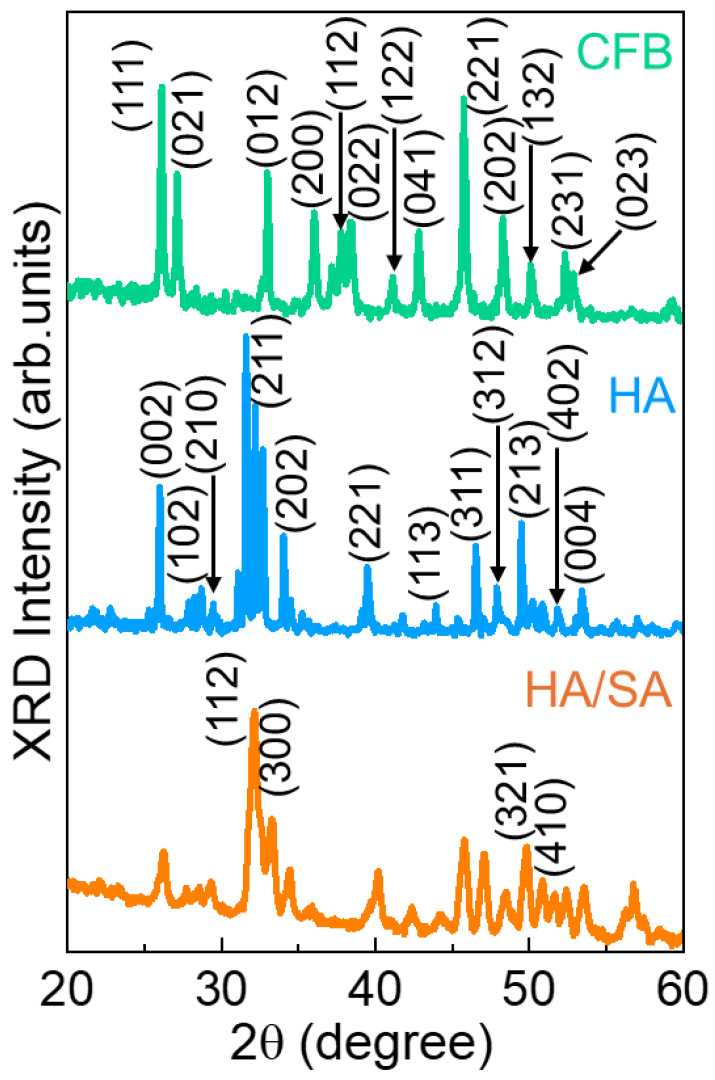
XRD pattern illustrating the crystal behavior of the CFB, HA, and HA/SA.

The analysis of the XRD crystalline orientation of the as-synthesized CFB, HA, and HA/SA composites are illustrated in Figure 2. The XRD measurements revealed the existence of calcium carbonate particles in the pure CFB samples that were treated with NaClO. The existence of aragonite microstructure with highly intense peaks is shown by the XRD pattern for the CFB. Hence, significant crystalline orientation is seen in naturally occurring CFB. A wide range of broad diffraction maxima in the 2θ region, which included 26.12° (111), 27.12° (021), 32.97° (012), 36.02° (200), 37.77° (112), 38.32° (022), 41.12° (122), 42.82° (041), 45.72° (221), 48.27° (202), 50.07° (132), 52.87° (231), and 53.97° (023), are highlighted in the XRD investigation that was associated with the calcium carbonate peaks of the as-derived CFB (JCPDS No.75-2230) [37,38]. Further, the hydrothermally produced HA nanoparticles exhibited the crystalline peaks at the 2θ range of 27.87° (002), 28.67° (102), 29.47° (210), 31.57° (211), 32.17° (112), 32.67° (300), 34.02° (202), 39.47° (221), 43.92° (113), 46.52° (311), 47.87° (312), 49.47° (213), 50.22° (321), 50.82° (410), 51.82° (402), and 53.42° (004), indicating the formation of a hexagonal phase structure of the HA (JCPDS 09-0432) [36,39]. The XRD pattern confirms that the prepared nanoparticles at 1200 °C showed the formation of a phase pure structure without secondary phase formation. Additionally, the pure crystalline HA phase of the calcium phosphates was produced through calcining at 1200 °C for two hours [35,40]. From the XRD pattern, it was clearly observed that the calcination process during the HA preparation led to the formation of carbonate-free composites.

In Figure 2, the XRD pattern for the HA/SA composite is displayed. The 2θ values for the HA/SA obtained from the XRD analysis are displayed at the peak positions of 26.23° (002), 28.57° (102), 29.37° (210), 32.14° (211), 33.24° (112), 34.41° (202), 40.20° (221), 42.37° (311), 47.05° (311), 48.49° (312), 49.86° (213), 51.66° (321), 52.40° (410), and 53.60° (402), confirming the presence of HA in the SA scaffold, which was well-matched with the standard data (No. 78-1420) [38,41,42]. Any further phase transition arrangement was not detected within the XRD pattern of the HA/SA scaffold. Furthermore, peak intensity decreased with peak width when adding SA into the HA structure. This intensity in peak reduction in the HA/SA scaffolds indicates that the polymer evenly covered the nanoparticle structure.

**Figure 3:**
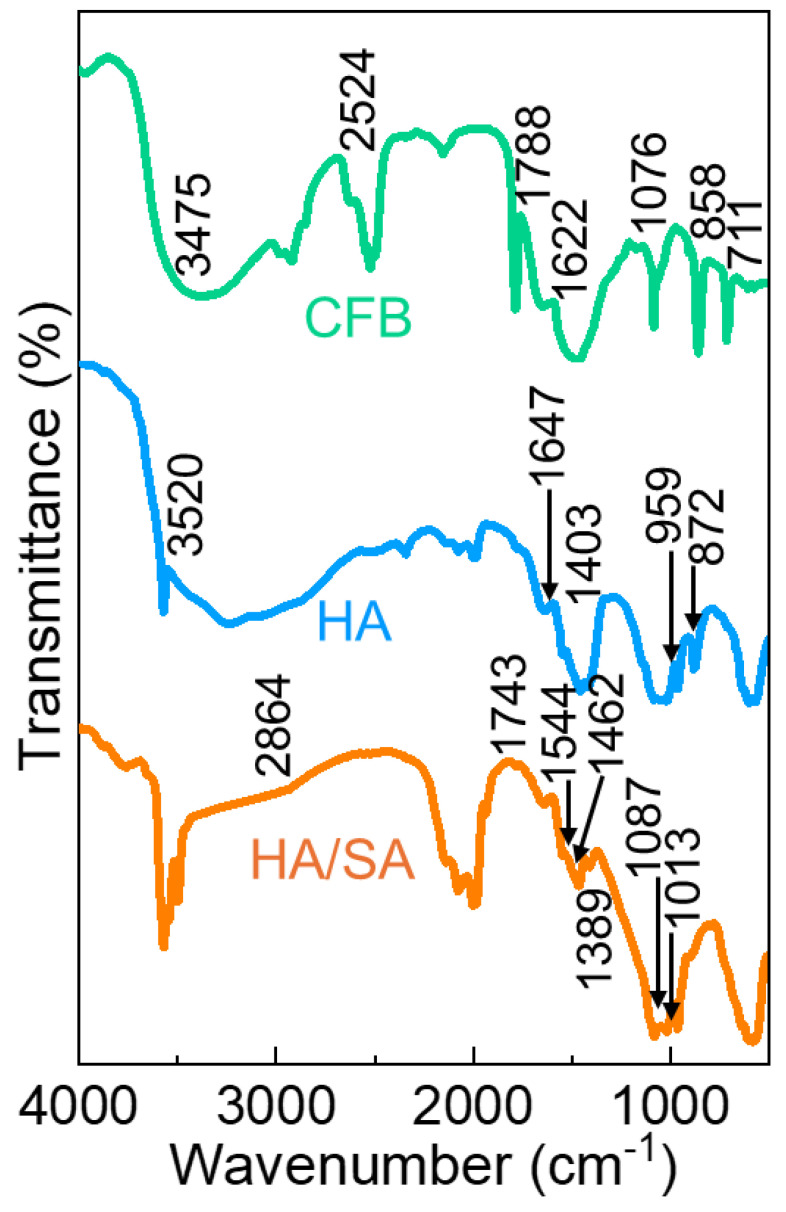
FTIR spectra of the CFB, HA, and HA/SA.

The structural changes occurring during the integration of the nanocomposite were investigated using FTIR spectroscopy. Figure 3 shows the FTIR spectra of the synthesized CFB, HA, and HA/SA scaffold. An assortment of peak intensities for the CFB that comprised the notable peaks at 2524, 1788, 1076, 858, and 711 cm^−1^ are illustrated in Figure 3. The mentioned peak values indicate the carbonate stretching and hydroxyl stretching of the CFB [38,43,44]. Similarly, the HA nanoparticles synthesized through hydrothermal treatment represent the vibrational spectrum at the wavenumber region at 1403, 959 and 872 cm^−1^ for the PO_4_^3−^ and trace amounts of CO_3_^2−^ ions. The absorbed atmosphere water molecules in HA were identified as responsible for of the notable peak elevation of bending and stretching vibrations exhibited at 3520 cm^−1^ and 1622 cm^−1^. From the IR image, it was observed that the as-prepared HA nanoparticles showed specific stretching and bending vibration peaks corresponding to the phosphate group. The hydrothermally treated HA samples showed the most significant vibrational peaks at around 1647, 1544, 1462, 1087, and 1013 cm^−1^ for the PO_4_^3−^ ions [38,41,45]. The FTIR spectrum further confirmed the transformation of highly carbonated CFB to hydroxyl substituted calcium phosphate derivative (HA) under the hydrothermal process.

The spectra obtained in the HA/SA scaffold synthesized from CFB are shown in Figure 3. The peaks found at 2864, 1734, 1629, 1389, 1254, and 1038 cm^−1^ demonstrated the presence of SA throughout the HA matrix. The mentioned peaks for the HA/SA scaffold were identified as the C–O–C, C–O, C–H, C=O, CO_3_^2–^, and C–H groups corresponding to the carbohydrate groups present in the SA polymer [42,46]. Additionally, the O-H signal for the composite with SA and polymer incorporation at the wavenumber region of 3520 cm^−1^ indicates an absorption of ambient water. These FTIR peaks suggest an intense relationship between the SA and the HA nanoparticles within their matrix. Consequently, the appearance of both the HA/SA scaffolds throughout the IR investigation considerably reveals the emergence of HA materials into the polymer matrix.

**Figure 4:**
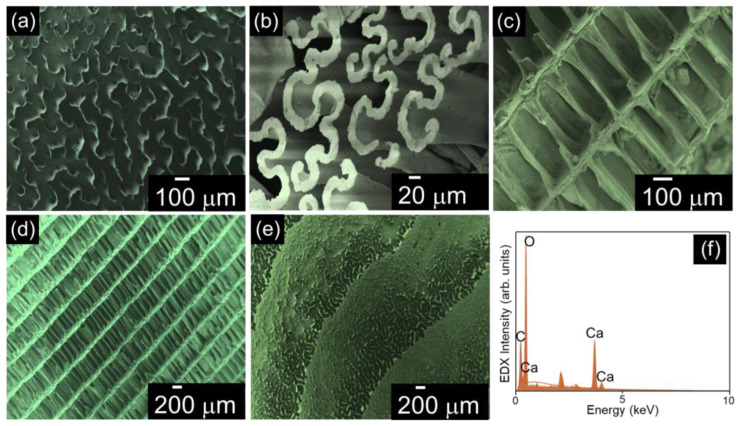
Surface analysis and different cross-sections of the CFB (**a**) sinusoidal profile; (**b**) developed sinusoidal profile; (**c**) channel formation; (**d**) layered channel arrangement (side view); (**e**) top view; and (**f**) EDX spectrum.

Figure 4a–f displays the FESEM micrographs and elemental analysis of the CFB. Figure 4a displays the CFB sample, which exhibited a sinusoidal shaped microstructure. After NaClO treatment, the hierarchical arrangement of CFB and its composition was determined. The sinusoidal profile of CFB showed the development of a pillar-like structure, as shown in Figure 4a,b, which was organized as a layered channel structure, as shown in Figure 4c–e. This clearly illustrates that the NaClO treatment did not affect the structural pattern of the CFB pattern. The EDS analysis for the CFB clearly demonstrates the presence of Ca, O, and C in its structure. From this elemental analysis, it was confirmed that the composition of the CFB was calcium carbonate, and the bone did not contain any other additional elemental composition in its structure.

**Figure 5:**
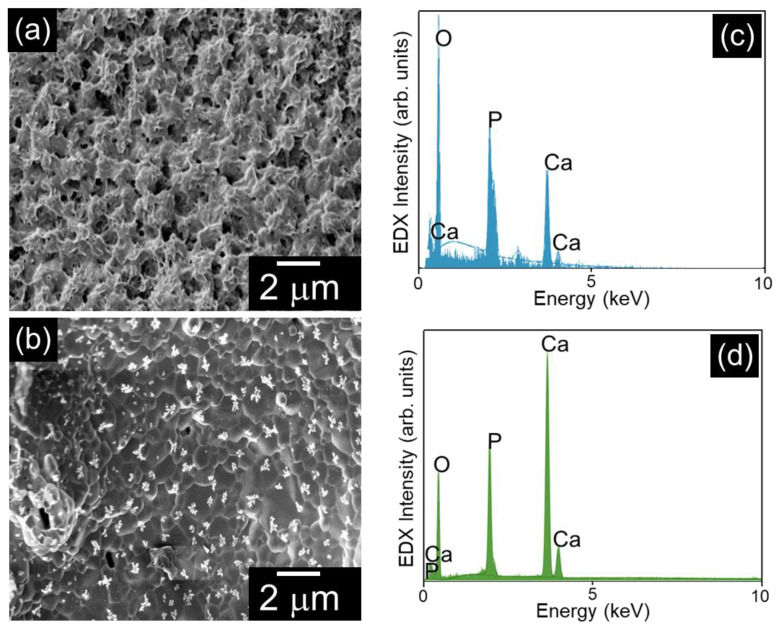
Morphological and elemental analysis of the hydrothermally prepared (**a**) HA and (**b**) HA/SA scaffold. EDX spectra of (**c**) HA and (**d**) the HA/SA scaffold.

Further, the SEM images clearly illustrate the gradual transformation of highly carbonated CFB into HA, as shown in Figure 5. The hydrothermal treatment of the HA led to the formation of highly porous structure formation (Figure 5a). The EDS analysis revealed the transformation of CO_3_^2−^ to PO_4_^-^ in the hydrothermally prepared HA sample prepared by the treatment of the CFB sample (Figure 5c). Thus, the observed surface modification and composition variation of HA obtained from CFB was similar to the XRD data. Further, the HA combined with SA is shown in Figure 5b. The SEM image of the scaffold indicates the formation of a nonporous structure without any cracks in the scaffold. Similarly, it also confirms the distribution of HA in the SA matrix during the formation of the HA/SA scaffold. Likewise, the EDS analysis revealed the presence of Ca, P, C, and O in its structure. The C atom in the EDS analysis was due to the incorporation of SA in the HA structure (Figure 5d).

**Figure 6:**
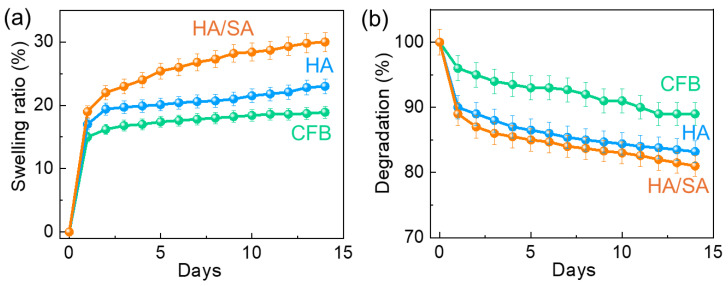
(**a**) Swelling ratio and (**b**) degradation behavior of the CFB, HA, and HA/SA scaffold in SBF for 14 days under humidified atmosphere.

In general, the swelling and degradation behaviors were investigated in artificially prepared stimulated body fluid to enhance the biomedical applicability of the as-developed biomaterials. Swelling tests were conducted using HA and HA/SA in SBF solutions to assess the biological characteristics of the as-developed CFB. Figure 6 displays the swelling behavior of the CFB, HA, and HA/SA composites. The findings showed that during the 14 days of SBF immersion, the swelling proportion improved to 17% for HA and 24% for the HA/SA scaffold, and afterwards, no significant modifications were identified.

Further, the determination of the physiological activity of the developed materials depends on the swelling behavior of composites that are performed. Therefore, the swelling feature of the composite improved the surface-to-volume ratio and stimulated improved viability properties throughout the particles for employing the nutrients acquired from SBF. Furthermore, Figure 6 demonstrates the degradation behavior associated with the as-developed CFB, HA, and HA/SA. According to the degradation behavior, the curve was evaluated and the degradation percentage within the as-developed HA/SA scaffold was significantly lower than that of the CFB and HA. Because of its excellent swelling and low degradation characteristics, the HA/SA scaffold, as created, has many advantages, resulting in this material suitable for long-term orthopedic applications.

**Figure 7:**
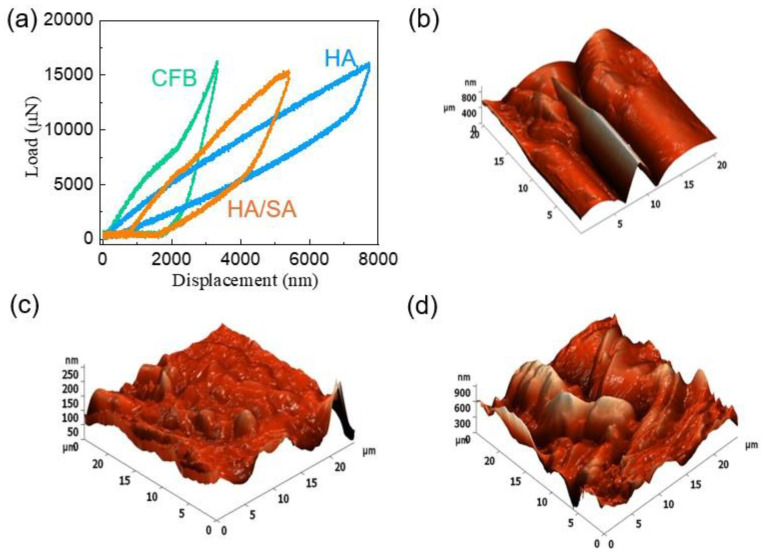
(**a**) Load vs. displacement graph and topographical analysis of the (**b**) CFB, (**c**) HA, and (**d**) HA/SA scaffold through nanoindentation.

The nanoindentation approach showed a considerable shift in the load–displacement curve at different loads (4–16 μN) in CFB, HA, and HA/SA scaffold, which is illustrated in Figure 7. The mechanical properties of the obtained scaffolds were determined, employing a nanoindentation investigation because of the considerable brittleness and anisotropic character of the CFB at various orientations. Figure 7 shows the curve of load and displacement for 16 µN after the sample scaffolds were exposed to 20 indents (2 µN to 40 µN). The hardness values of the hydrothermally processed CFB, HA, and HA/SA scaffold were determined as follows: 0.15 GPa, 0.14 GPa, and 0.24 GPa with a force applied of 4 µN. Young’s modulus values were determined to be 4.14 GPa, 4.65 GPa, and 5.34 GPa, according to the prepared samples. Further, the determined hardness and Young’s modulus values are tabulated in Table 1. The indentation curves reveal that integrating SA into the HA scaffold resulted in the highest displacement with a consistent load, which indicates that the values of toughness and Young’s modulus increased.

Employing the nanoindentation test, the Young’s modulus and hardness evolution at maximum load for the HA and HA/SA composites were determined. The prepared composite hardness and Young’s modulus values were determined and are displayed in Figure 7. Comparing the average hardness value of the CFB to that of the HA and HA/SA scaffolds at the maximum load of 16 μN revealed variations. There was a noticeable shift in the mechanical integrity as indicated due to the loading behavior exhibited by the indentation curve during SA incorporation in the HA scaffold. In accordance with the indentation graph, the inclusion of SA in the HA structure was seen to exhibit no fractures, splits, or shifts in slope. The HA and HA/SA had comparatively greater hardness values than the CFB, and this may be attributed to the SA inclusion into the HA structure. The increasing hardness confirmed that the scaffold facilitated the development of promising materials for improved biomedical applications. The observations indicate that the HA and the scaffold prepared with SA had higher hardness and Young’s modulus values. However, the HA showed fractures while the load was raised for 16 µN; instead, the HA samples did not indicate such structural changes. From this, it was revealed that the mechanical behavior of the HA/SA scaffolds can tolerate an applied load of 16 µN.

**Figure 8:**
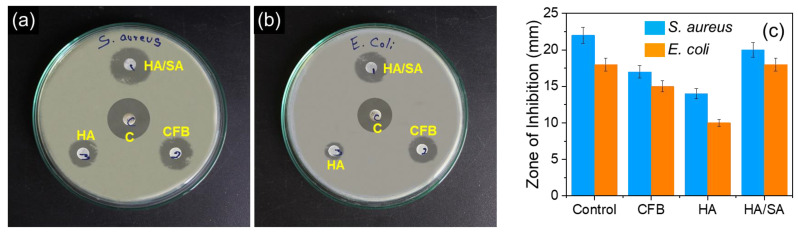
Photographic image of antibacterial resistance of the CFB, HA, and HA/SA scaffold against (**a**) *E. coli*, (**b**) *S. aureus*, and (**c**) Bar graph for zone of inhibition vs. Samples.

*E. coli* and *S. aureus* typically constitute the primary pathogens that develop infections in wounds, which lead to the secondary procedure of the recovery. Therefore, the disc diffusion method was used to examine the antibacterial activity against the pathogenic microorganisms. Figure 8 displays the dimensions of the zone of resistance for the CFB, HA, and HA/SA compounds towards *E. coli* and *S. aureus*, and the control used in this analysis was chloramphenicol. Generally, CFB bone exhibited the bacterial resistance of 17, and 15 mm indicated the clear zone formation for the E. coli and S. aureus. HA possessed clear zone formation of 14 and 10 mm for the bacterial strains. The higher bacterial resistance for the CFB bone was maybe due to the collagen present in the biogenic waste. Further, the higher temperature sintering removed the polymeric substance from the CFB bone, which will reduce the bacterial resistance for the HA nanoparticles. Hence, the prepared HA nanoparticles revealed reduced resistance to the bacterial strains. Additionally, the antibacterial activity of the HA/SA scaffold demonstrated a greater zone of inhibition development of 20 and 18 mm *E. coli* and S. aureus. HA and HA/SA demonstrated a greater inhibitory zone against *E. coli* in comparison with *B. subtilis*. According to studies, the introduction of polymer with antibacterial qualities into HA may be able to prevent the development of microorganisms. The SA combined with HA in the composite indicates an enhanced activity. Therefore, HA/SA demonstrated superior antibacterial effectiveness against Gram-negative *E. coli* within the composites.

**Figure 9:**
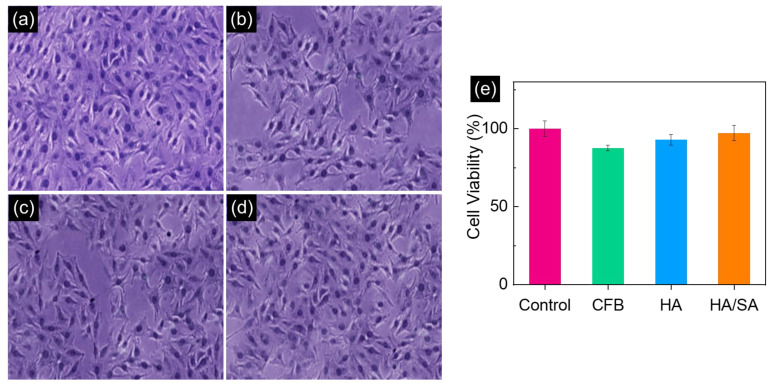
Optical microscopy images of the (**a**) control, (**b**) CFB, (**c**) HA, and (**d**) HA/SA scaffold against the MG 63 cell line, and (**e**) the cell viability of the samples.

The MTT assay used to analyze the cell viability of CFB, HA, and HA/SA nanocomposite scaffolds is represented in Figure 9. The obtained results showing an optical microscopic image at the concentration of 50 µL and a bar diagram of the viable cells after 72 h of incubation are displayed in Figure 9. Generally, the toxicological behavior of nanomaterials depends on the cellular uptake of nanoparticles, intracellular distribution, exocytosis, particle size, crystalline shape, aspect ratio, and surface functionality of nanoparticles. These properties are favorable for the cell viability, which is toxicological behavior of nanoparticles smaller than macro- and microparticles. In comparison with cell culture analysis, the obtained result indicates the major presence of CaCO_3_ in the pure CFB showed a minimum cell viability of 87.6%, whereas hydrothermally treated CFB calcined at high temperature majorly contained HA, which gradually increased the cell viability to 92.8% and exhibited non-toxicity to the proposed cell culture. Further, the HA/SA derivates showed excellent cell viability of 97.2%, which indicates the enhanced biocompatibility of the prepared nanocomposites that were potentially beneficial to the bone-related applications. The pure CFB showed minimal cell viability, whereas HA and HA/SA illustrate significant enhancement in the cell viability. However, the high cell viability was observed in HA/SA scaffolds, which was maybe due to the specific anionic bond, electrical stimulation, and the gelling structure of the SA present in the HA structure. These results clearly confirm that the improved form of a crystalline and morphological structure of nanocomposites provided excellent biocompatibility and dispersion ability from the cell shape completely spread out, showing a nice cell-grown environment. This investigation confirms that the MG-63 cell viability on the hydrothermally modified HA/SA scaffolds was higher than the CFB and HA.

## 4. Conclusions

The lamellar region of cuttlefish bone was hydrothermally transformed into hydroxyapatite, a bone-forming mineral. The XRD and FTIR analysis clearly illustrated the gradual phase transformation of calcium carbonate to hydroxyl apatite under hydrothermal process conditions of 180 °C for 48 h, followed by sintering at 1200 °C. Also, the hydrothermal treatment did not affect the unique hierarchical arrangement of cuttlefish bone. The mechanical behavior of the hydrothermally prepared HA increased with the addition of SA. Furthermore, this investigation revealed that the HA/SA nanocomposite exhibited potential antibacterial activity at the concentration of 50 µL against different bacterial pathogens such as *E. coli* and S. aureus. The in vitro cell viability with the human osteosarcoma (MG 63) cell line at the concentration of 50 µg/mL was analyzed, and the observed results show that the HA/SA nanocomposite revealed an excellent cell viability of 97.2% compared with the CFB and HA nanoparticles. In conclusion, the hydrothermally synthesized HA/SA nanocomposite from the biogenic waste was highly suitable for bone tissue regeneration application with enhanced biological performance.

## Figures and Tables

**Table 1:** Nanoindentation study on CFB, HA, and HA/SA scaffolds.

S. No.	Samples	Hardness (GPa)				Young’s Modulus (GPa)			
		4 µN	8 µN	12 µN	16 µN	4 µN	8 µN	12 µN	16 µN
1.	CFB	0.15	0.30	0.09	0.16	4.14	3.87	2.00	2.51
2.	HA	0.16	0.28	0.15	0.17	4.65	3.15	4.28	2.83
3.	HA/SA	0.24	0.30	0.15	0.19	5.34	3.72	3.13	5.60

## Data Availability

Data is contained within the article.
